# Allergic Aspects of IgG4-Related Disease: Implications for Pathogenesis and Therapy

**DOI:** 10.3389/fimmu.2021.693192

**Published:** 2021-07-07

**Authors:** Despina Michailidou, Daniella Muallem Schwartz, Tomas Mustelin, Grant C. Hughes

**Affiliations:** ^1^ Division of Rheumatology, University of Washington, Seattle, WA, United States; ^2^ National Institute of Allergy and Infectious Diseases, National Institutes of Health, Bethesda, MD, United States

**Keywords:** IgG4-related disease, type 2 immmune response, allergy, eosinophils, basophils, mast cells, alarmins

## Abstract

IgG4-related disease (IgG4-RD) is a rare systemic fibroinflammatory disease frequently associated with allergy. The pathogenesis of IgG4-RD is poorly understood, and effective therapies are limited. However, IgG4-RD appears to involve some of the same pathogenic mechanisms observed in allergic disease, such as T helper 2 (Th2) and regulatory T cell (Treg) activation, IgG4 and IgE hypersecretion, and blood/tissue eosinophilia. In addition, IgG4-RD tissue fibrosis appears to involve activation of basophils and mast cells and their release of alarmins and cytokines. In this article, we review allergy-like features of IgG4-RD and highlight targeted therapies for allergy that have potential in treating patients with IgG4-RD.

## Introduction

IgG4-related disease (IgG4-RD) is a rare, potentially life-threatening fibro-inflammatory disease of unknown etiology. IgG4-RD is clinically characterized by the presence of fibro-inflammatory masses in various organs, dense lymphoplasmacytic infiltration of IgG4-postitive plasma cells, storiform fibrosis and elevated serum levels of IgG4 (1, 2). Although abnormal adaptive immune responses are thought to play a major role in the development of IgG4-RD, the specific mechanisms are incompletely understood. Of note, IgG4-RD patients often suffer from allergic diseases such as asthma, rhinosinusitis, rhinoconjunctivitis or skin diseases such as atopic dermatitis (3–5).

Allergy describes an abnormal adaptive immune response to foreign antigen (allergen). Allergens are an important driver of Type 2 immune responses, which likely evolved in response to helminth infections (6). The hallmark of clinical allergy is the development of allergen-reactive T helper 2 (Th2) cells, which produce Type 2 cytokines (IL-4, IL-5, IL-13) that promote Ig isotype switch in B cells. This results in production of allergen-specific IgE, which then activates mast cells and basophils to release Type 2 cytokines and preformed inflammatory mediators (7). Allergic inflammation can also be driven by epithelial-derived alarmins like thymic stromal lymphopoeitin (TSLP) and IL-33, which trigger allergen-independent production of Type 2 cytokines (8–12). These cytokines recruit effectors like mast cells, basophils, type 2 innate lymphoid (ILC-2) cells and eosinophils (13, 14). Type 2 effectors perpetuate the allergic inflammatory response and ultimately promote tissue damage. For example, IL-5 is particularly important for eosinophil activation, whereas IL-13 drives airway fibrosis (15).

IgG4-RD and allergic disorders share certain immunopathologic features, such as activation of Th2-like pathways, elevated IgE and IgG4 production, peripheral blood eosinophilia, and tissue mast cell infiltration (5, 16, 17). Importantly, clinical and epidemiologic evidence suggests that IgG4-RD and allergic entities are not manifestations of a single disease but rather distinct disease processes that both involve type 2 immune activation (18–20), overproduction of pro-Th2 cytokines, and the development of IgE- and IgG4-secreting B cells (21, 22). The pathogenesis of IgG4-RD remains unknown, and effective medical therapies are limited. In this review article, we discuss shared etiopathogenetic mechanisms and clinical associations of IgG4-RD with allergic diseases. We then explore potential implications for diagnosis and management of patients with IgG4-RD.

## Immuno-Pathophysiological Mechanisms in IgG4-RD

**Figure 1:**
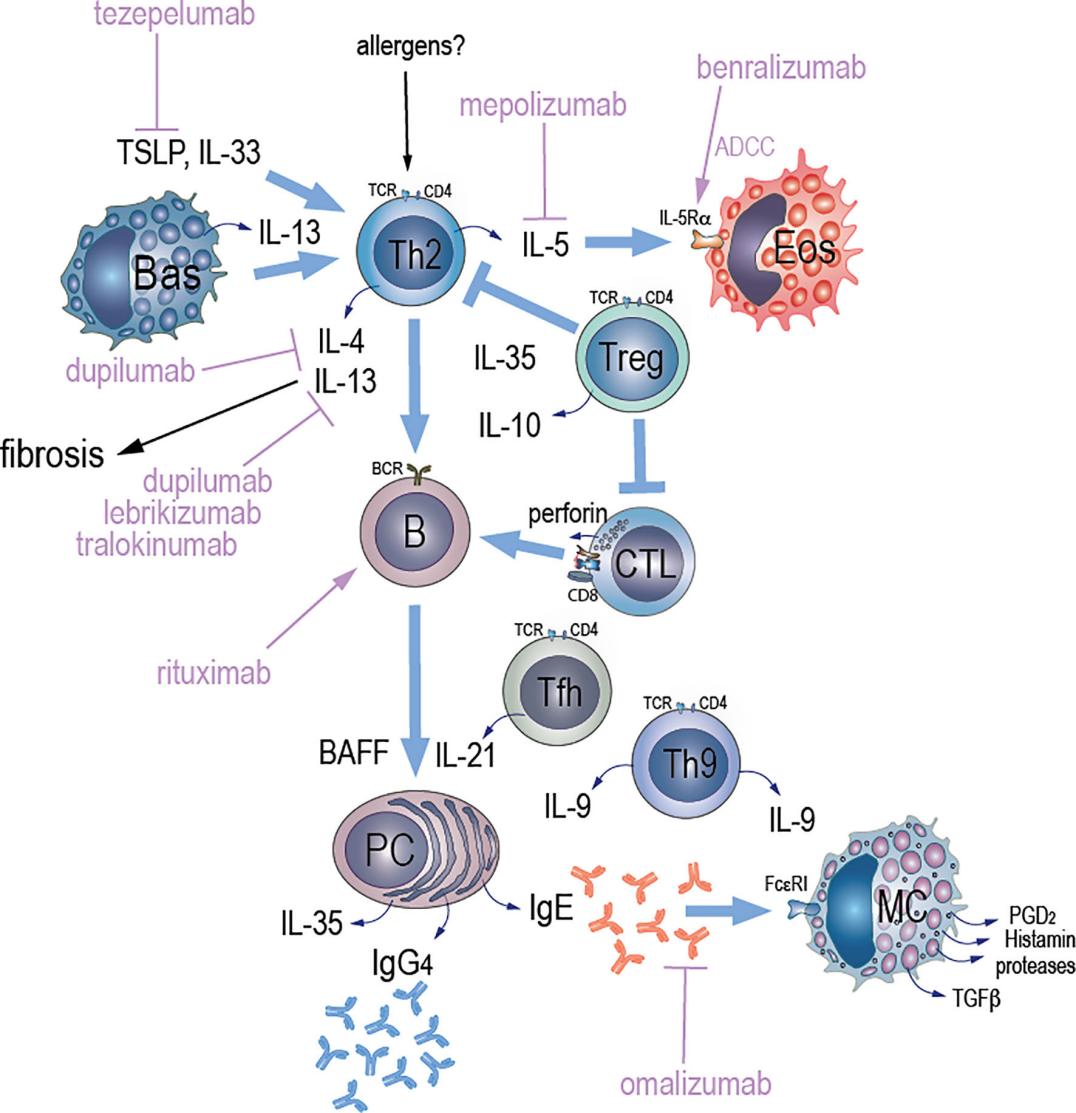
Potential mechanisms of Type 2 immunity and therapeutic targets in IgG4-RD. We hypothesize that an unknown antigenic stimulus (allergen) triggers Th2 cells and activates them to secrete interleukins. Activation of Th2 cells by TSLP promotes secretion of IL-4, IL-5, and IL-13, which activate B cells and eosinophils. Both TSLP and IL-33 may contribute to IgG4 accumulation via induction of a Th2 cytokine environment. TLR-activated basophils secrete BAFF and IL-13. BAFF that is also secreted from B cells promotes immunoglobulin class switching while IL-13 maintains Th2 cell-dominant immune responses contributing to increased IgG4 production. A population of effector memory CD4+ T cells with a cytotoxic function (CD4+ CTLs) that arises from chronic antigenic stimulation has also been described in IgG4-RD. An antigen-driven process that requires an interaction between CD4+ CTLs and activated B cells that serve as antigen presenting cells might be implicated in the pathogenesis of igG4-RD based on observations of significant reduction of circulating CD4+CTLs and plasmablasts after glucorticoid therapy or B cell depletion with rituximab through antibody-dependent cell-mediated cytotoxicity (ADCC). Other key players in the pathogenesis of IgG4-RD include follicular CD+T helper (Tfh) cells that induce IgG4 class-switching, expansion of plasmablasts, and production of autoantibodies. Tfh cells drive immunoglobulin class switching and promote ectopic GC formation through IL-21 production. IL-10 that is secreted by T regulatory (Treg) cells drives the differentiation of IgG4-class-switching B cells to IgG4-secreting plasma cells, whereas IL-35 may suppress inflammation via activation of effector Tregs and suppression of CD4+CTLs. Plasma cell derived IL-35 may also drive the differentiation of naïve CD4 T cells towards a Th9 phenotype, and IL-9 release, which further promotes plasma cell differentiation and IgG4 immunoglobulin class switching. IgE secreted by plasma cells stimulates mast cells via its binding to the high-affinity IgE receptor (FcεRI) leading to release of granule contents and cytokines, which together drive collagen production and fibrosis. Targeting of TSLP-mediated signaling pathway with tezepelumab, and resultant abrogation of Th2 cascades, might be one of potential therapeutic options in IgG4-RD. Blocking both IL-4 and IL-13 signaling pathways with dupilumab might reduce inflammation and fibrosis in igG4-RD. Blockage of IL-13 signaling pathway that is implicated in Th2-related fibrosis with either lebrikizumab or tralokinumab might be another attractive therapeutic target in igG4-RD. Depletion of IL-5R-expressing eosinophils through ADCC with benralizumab or blockage of IL-5 with mepolizumab might reduce eosinophilia and could be an alternative therapeutic targets in patients with IgG4-RD that have peripheral or tissue eosinophilia. Dissociation of pre-bound IgE from FcεRI with omalizumab might reduce activation of mast cells and production of TGF-β1 that induces fibrosis.

The pathogenesis of IgG4-RD is not yet fully understood, but it is thought to involve varied mechanisms including autoimmunity-associated and allergy-associated factors (**Table 1**). Patients with IgG4-RD classically have oligoclonal expansion of both B and T cells in the peripheral blood, with oligoclonally expanded CD4+ T lymphocytes in the affected tissues (23). CD4+ T lymphocytes expressing CD40 ligand (CD40L) drive differentiation of plasmablasts, germinal center formation (GC), and production of IgG4 (24). A population of effector memory CD4+ T cells with a cytotoxic function (CD4+ CTLs) has also been described in IgG4-RD and it is possible that arises from chronic antigenic stimulation. An antigen-driven process that requisites an interaction between CD4+CTLs and activated B cells that serve as antigen presenting cells might be implicated given the significant reduction of circulating CD4+CTLs and plasmablasts after glucorticoid therapy (**Figure 1**) (25). CD4+CTLs also express IL-6, TGF-β, and IFN-γ, which may contribute to chronic inflammation and fibrosis in IgG4-RD (26, 27). On the other hand, patients with severe asthma have reduced IFN-γ production, which promotes differentiation of CD4+T-cells into Th2 cells (28) (**Table 1**).

**Table 1:** Mediators of innate and adaptive immunity in IgG4-RD and allergic disorders.

Mediators of innate and adaptive immunity	Roles in IgG4-RD	Roles in Allergic disorders
**Memory CD4+ T cells**	Secretion of profibrotic cytokines (e.g., IL-6, IFN-γ and TGF-β) promoting induction of IgG4 class-switching, expansion of plasmablasts, and production of autoantibodies (4, 26, 37, 38). Secretion of IL-4, IL-5, IL-13 promoting eosinophil infiltration (60)	Secretion of pro-allergic cytokines, (IL-4, IL-5, and IL-13) promoting both immunoglobulin E (IgE)–and eosinophil mediated immune responses (21)
**T follicular helper cells**	Promotion of isotype class switching to IgE and IgG4, and ectopic GC formation through IL-21 production (23, 30)	Secretion of IL-4 and promotion of isotype switching to IgE (29)
**B cells**	Activation of B cells results in class switching from IgM to IgE and/or IgG4 (56)	Activation of B cells results in the production of IgG, IgA, and IgE antibodies in allergy (58)
**Mast cells**	Mast cell activation by high-affinity FcϵRI receptor crosslinking might contribute to fibrosis in IgG4-RD *via* degranulation and release of several granule-associated molecules (e.g., histamine) and TGF-β (54, 55).	Type I hypersensitivity allergic reactions, are mediated by cross-linking of antigen-specific IgE immune complexes and FcϵRI receptors on the membrane surface of mast cells (106, 107)
**Basophils**	TLR2 and/or TLR4-activated basophils may promote IgG4 production *via* TLR signaling (69)	Basophils produce cytokines, such as IL-4 and IL-13, and release histamine and leukotriene after activation of FceRI by IgE crosslinking (63)
**Eosinophils**	Eosinophils may contribute to IgG4-RD pathogenesis by inducing fibrosis *via the* production of TGF-β and IL-13 (66)	Eosinophils produce IL-13, TGF-β that are involved in the pathogenesis of allergic diseases. Eosinophils also directly activate mast cells (145)
**IgG1**	Decreased complement levels in IgG4-RD may involve complement fixation by IgG1-containing immunocomplexes (53) Antagonistic function of IgG4 antibodies against pathogenic IgG1 autoantibodies noted when injected from a patient with autoimmune pancreatitis into neonatal mice and immunostaining in pancreatic tissue was performed (55)	Majority of IgE+ cells arise from somatically hypermutated IgG1-expressing cells as demonstrated from analysis of Ig heavy regions in peripheral blood of patients with allergy (50) Binding of IgG1-containing immunocomplexes to the IgG inhibitory Fc gamma receptor (FcγRIIB) suppresses complement C5aR-mediated inflammatory signaling in allergy (54)
**IgG4**	Pathogenic role in IgG4-RD (1, 2)	Thought to promote tolerance in the context of food allergy (45)
**IgE**	IgE-positive mast cells might contribute to fibrosis in IgG4-RD, given their presence in IgG4-related fibrosclerotic mesenteric masses (5)	IgE sensitizes mast cells to release biologically active mediators such as histamine and prostaglandins in an antigen-specific manner in allergic diseases (43)
**IFN-gamma**	IFN-γ may contribute to chronic inflammation and fibrosis in IgG4-related dacryoadenitis and sialoadenitis (27)	Patients with severe asthma develop significantly reduced IFN-γ production in response to allergens that may result in increased IgE production by either switch of B cells into IgE producing plasma cells or differentiation of CD4+T-cells into Th2 cells (28)
**Type 2 cytokines (IL-4, IL-5, IL-13)**	Type 2 cytokines are up-regulated in the tissue of IgG4-RD, promoting peripheral blood eosinophilia and activating B cells to class switch from IgM to IgE and/or IgG4 (57–59)	Type 2 cytokines recruit effectors like mast cells, basophils, ILC-2 cells and eosinophils in allergy (12) Type 2 cytokines promote Ig isotype switch in B cells, resulting in production of allergen-specific IgE (7)
**Alarmins (TSLP, IL-33)**	Alarmins may contribute to chronic inflammation and IgG4+ B cell accumulation *via* induction of mucosal Type 2 immunity (10, 60, 72)	Alarmins drive allergic inflammation by triggering Type 2 cytokines (8–12)

IL-6, interleukin 6; IFN-γ, interferon gamma; TGF-β, tumor growth factor beta; IL-4, interleukin 4; IL-5, interleukin 5; IL-13, interleukin 13; GC, germinal center; IL-21, interleukin 21; IgE, immunoglobulin E; IgM, immunoglobulin M; IgG4, immunoglobulin G subtype 4; IgA, immunoglobulin A; TLR, toll like receptor; FcγRIIB, Fc gamma receptor IIB; C5aR, complement 5a receptor; ILC-2, type 2 innate lymphoid cells; IgG4-RD, IgG4-related disease; TSLP, thymic stromal lymphopoeitin; IL-33, interleukin 33; Th2, T helper 2 cells.

Both memory and follicular CD4+ T helper cells are thought to contribute to IgG4-RD *via* secretion of profibrotic cytokines (e.g., IL-6, IFN-γ and TGF-β), induction of IgG4 class-switching, expansion of plasmablasts, and production of autoantibodies (4). T follicular helper (Tfh) cells have a particularly important role in allergy by promoting isotype switching to IgE *via* secretion of IL-4 (29) and in IgG4-RD by contributing to isotype class switching and promoting ectopic GC formation through IL-21 production (**Figure 1** and **Table 1**) (23, 30). IL-21 combines with T regulatory cell (Treg)-derived IL-10 and myeloid-derived IL-12 to promote IgG4 production (31–33). IL-10 has been specifically described as a key cytokine that promotes the differentiation of IgG4-switched B cells to IgG4-secreting plasma cells (34) (**Figure 1**). IL-10 production can be enhanced by IL-27 by converting activated inflammatory CD4+T cells into IL-10 producing Treg cells (35). Immunohistochemically CD4+CD25+Foxp3+Treg cells were more frequently found to infiltrate lesions in autoimmune pancreato-cholangitis compared to primary sclerosing cholangitis and primary biliary cirrhosis, and produced more IL-10 and TGF-β as determined by RT-PCR (18). Massive expansion of polyclonal IgG4-switched B and/or plasma cells appears to drive tissue infiltration and organ damage in IgG4-RD (36). Activation of B cells in GCs results in production of B cells with high-affinity antigen receptors, and in generation of plasmablasts. It is possible that these plasmablasts induce activation of CD4+ T cells and result in secretion of cytokines, such as IL-1, TGFβ1, IFN-γ and other signaling mediators of the innate and adaptive immunity driving the fibrotic pathology in igG4-RD (37, 38) (**Table 1**).

These B cell subsets express CD20, so this is perhaps why B cell depletion with the anti-CD20 monoclonal antibody rituximab is effective for many forms of IgG4-RD (39). The mechanisms of action appear to include depletion of antigen-presenting B cells, subsequent reduction in activation of pathogenic CD4+ T cells and their release of inflammatory and profibrotic cytokines (4). Response to rituximab therapy correlates with reduction in frequency of distinct circulating plasmablast clones; and relapse corresponds with their return. This suggests that relapse after B cell depletion involves recruitment and activation of naïve B cells and/or re-emergence of memory B cells resistant to rituximab (40).

## Type 2 Immunity in IgG4-RD

Amongst the factors thought to contribute to IgG4-RD, Type 2 immunity has been proposed to play a significant role in disease pathogenesis. This includes the hallmark Type 2 immunoglobulin IgE, effector cells such as mast cells and Th2 cells, and cytokines/chemokines. Much of the data suggesting a role for Type 2 factors in IgG4-RD are observational, and a direct mechanistic link has not yet been established for these factors. Additionally, it is important to note that Type 2 factors are not the only contributors to IgG4-RD pathogenesis and that other factors including IFN-γ and IgG1 are also likely to have an important role (**Table 1**). However, the large amount of data demonstrating the presence of Type 2 factors in the circulation and tissue of IgG4-RD patients, and the correlation of these factors with clinical features, suggest that Type 2 immunity has a role in IgG4-RD.

### The Role of IgE in IgG4-RD

Allergen-specific IgE is a major mediator of clinical allergy, and other types of IgE such as autoreactive IgE are known to play important roles in the development of certain autoimmune diseases (41, 42). IgE sensitizes mast cells to release histamine and prostaglandins in an antigen-specific manner in allergic diseases (43) (**Table 1**). In parallel to the IgE response, there is also production of high levels of allergen–specific IgG4 antibodies the potential role of which has not yet been clearly established in allergy (44). A recent study showed that there was a higher overlap between IgE and IgG4 cow milk (CM) epitopes in patients tolerating CM compared to those who reacted to it, suggesting a blocking role of IgG4 and the importance of balance between IgE and IgG4 in tolerance (45).

IgE may also play a major role in IgG4-RD, as a significant correlation between serum IgE and IgG4 levels has been observed in patients with concurrent diagnoses of IgG4-related Type 1 autoimmune pancreatitis and clinical allergy (46). In the context of clinical allergy, repeated exposure to certain allergens promote a “modified allergy” milieu in which Th2-derived IL-4 and Treg-derived IL-10 combine to induce IgG4 production (47). It remains to be determined whether these mechanisms underlie IgG4-dominant responses in IgG4-RD, and how other IgG4-promoting cytokines like IL-21 may contribute (**Table 1**). This is in part because there is a paucity of information about Ig levels before onset of IgG4-RD.

The exact source of the elevated IgG4 and IgE in the blood and tissue in IgG4-RD patients is unknown. Tissue IgE-positive memory B cells and plasma cells may emerge directly from a germinal center or through indirect class switching from other intermediate antibody isotypes such as IgG1 to IgG4 (48, 49). Looney et al. found evidence that the majority of IgE+ cells derive from somatically hypermutated IgG1-expressing cells as demonstrated from analysis of Ig heavy regions in peripheral blood of patients with allergy and indirect isotype switching from IgG4 to IgE contributes to the IgE pool (50) (**Table 1**). In addition, IgG4+ B cells may bind IgE molecules *via* their low-affinity FcϵRII receptors (51). The importance of this observation remains to be determined but suggests integration of IgE and IgG4 responses at the B cell level. Moreover, mast cells infiltrating IgG4-RD tissue show strong cytoplasmic staining for IgE *via* its binding to the high-affinity IgE receptor (FcϵRI) (52, 53). IgE-positive mast cells may contribute to fibrosis in IgG4-RD, which is supported by their presence in an IgG4-related fibrosclerotic mesenteric masses; in these cases, serum IgE levels could be a useful biomarker in diagnosis and prediction of disease relapse (5). Activation of mast cells by high-affinity FcϵRI receptor crosslinking might contribute to fibrosis in IgG4-RD *via* degranulation and release of several granule-associated molecules (e.g., histamine) and TGF-β, that together may drive fibroblast activation, collagen production, and tissue fibrosis (54, 55), (**Table 1** and **Figure 1**). However, the precise role of mast cells in IgG4-RD tissue fibrosis remains to be determined.

### Type 2 Immune Effector Cells in IgG4-RD

Although IgE is a hallmark of clinical allergy, Th2 cells and their associated cytokines are considered major drivers of Type 2 immune responses (56). It is thought that in IgG4-RD these Th2 cytokines promote peripheral blood eosinophilia and activate B cells to class switch from IgM to IgE and/or IgG4, as they do in allergic diseases (57–59) (**Table 1** and **Figure 1**). Indeed, in the peripheral blood and involved tissues of patients with IgG4-related dacryoadenitis and sialadenitis, there was a significant increase in stimulation expressed gene 2 (ST2) memory-positive Th2 cells that were characterized by high IL-5 expression (60). Moreover, the Th2 cytokines IL-4, IL-5, and IL-13 were found to be up-regulated in the tissue of IgG4-related sclerosing pancreatitis and cholangitis (18). Thus, chronic Th2 activation may contribute to IgG4-RD-associated tissue phenotypes such as eosinophil infiltration and fibrosis (61).

Effector cells like eosinophils and basophils also can directly mediate type 2 immune responses (62, 63). These cells are generally recruited by and expand in response to Type 2 cytokines like IL-5, although in some Type 2 immune syndromes they are constitutively dysregulated (64). Eosinophilia has been described in patients with IgG4-RD with or without allergy (65). Eosinophils may contribute to IgG4-RD pathogenesis by inducing fibrosis *via* the production of TGF-β and IL-13 (66) (**Table 1**).

Basophils are Type 2 effectors that both respond to and promote Th2-mediated inflammation (63). Toll like receptor (TLR) activation of basophils from patients with IgG4-RD led to release of B-cell-activating factor (BAFF) and IL-13, enhancing IgG4 production by B-cells from healthy controls (67). BAFF promotes immunoglobulin class switching, whereas IL-13 maintains Th2-cell dominant immune responses (68) (**Figure 1**). Thus, activated basophils may induce IgG4 production *via* TLR signaling (69) (**Table 1**). The specific TLR ligands that activate basophils in IgG4-RD patients are unknown.

### Type 2 Cytokines and Chemokines in IgG4-RD

In addition to Type 2 cytokines like IL-4 and IL-13, Type 2 immune responses can also be induced by alarmins, which are epithelial-derived immune activating proteins released in response to cellular stress, injury, or death (70). IL-33 and thymic stromal lymphopoietin (TSLP) are two alarmins that are critical for promoting acute Type 2 immune responses at barrier surfaces (71). Both TSLP and IL-33 were found to be up-regulated in the submandibular glands of patients with IgG4-RD; these alarmins may contribute to chronic inflammation and IgG4+ B cell accumulation *via* induction of mucosal Type 2 immunity (10, 60, 72).

Type 2 chemokines such as thymus and activation-regulated chemokine (TARC) can also be important mediators of allergic disease (73). TARC is a ligand for the C-C chemokine receptors CCR4 and CCR8, which are predominantly expressed by Th2 cells (74). High serum levels of TARC were observed in patients with IgG4-RD. TARC levels did not correlate with blood IgG4 levels or peripheral eosinophil counts, but correlated positively with multiple organ involvement and could be a new therapeutic target of IgG4-RD (75).

IL-35, a cytokine of the IL-12 family appears to suppress airway inflammation in asthma (76, 77). The role of IL-35 in IgG4-RD remains unclear, as potentially it could play protective or pathogenic roles. For example, both plasma and pancreatic tissue levels of IL-35 were elevated in IgG4-related type 1 autoimmune pancreatitis (AIP) and appeared to suppress inflammation *via* activation of effector Tregs and suppression of Th2 immune responses (78). However, in another study, IL-35 was found to be secreted by IgG4-positive plasma cells in the plasma of patients with hepatobiliary and/or pancreatic involvement of IgG4-RD, driving the differentiation of naïve CD4 T cells towards Th9 cells *via* an interferon regulatory factor 4 (IRF4) signaling pathway. This differentiation led to secretion of IL-9 from Th9 cells that promoted plasma cell differentiation and immunoglobulin class switch towards IgG4 (79). IL-9 is also known to up regulate mast cell IgE receptor expression, leading to increased IgE production and inducing eosinophil growth (80) (**Figure 1**). Thus, IL-35 might contribute to an important amplification loop in IgG4-RD that could make it a potential therapeutic target in IgG4-RD.

### Genetic Overlap of IgG4-RD With Type 2 Immunity

The genetic landscape of IgG4-RD is not well explored. Genetic studies of IgG4-RD identified two susceptibility loci, the human leukocyte antigen (HLA)-DRB1 and Fc gamma receptor 2B (FCGR2B) (81). Interestingly, the HLA-DRB1 risk allele was also associated with peanut allergy, consistent with the idea of shared immunopathogenesis between allergy and IgG4-RD (82). In addition, a functional single nucleotide polymorphism (SNP) in the inhibitory FCGR2B was found to be associated with atopy and increased IgE production, suggesting that FCGR2B SNP may have a role in allergy and IgG4-RD *via* its effects on IgE production (83).

## Allergic and Type 2 Immune Conditions Associated With IgG4-RD

### Association of IgG4-RD With Asthma and Allergic Rhinitis

A cross-sectional study of 114 patients with IgG4-RD showed that 19% had been previously diagnosed with allergic diseases, including allergic rhinitis, sinusitis, or bronchial asthma (84). In a retrospective study of 64 patients with IgG4-RD, the prevalence of allergic rhinitis was significantly higher compared to that in patients with Sjogren’s syndrome (26% vs 2%, p<0.001) (85). In another retrospective observational study of 31 patients with orbital IgG4-RD, asthma was found in 52% (86). A case of IgG4-RD with bronchial asthma was also reported (87). Adult onset asthma and periocular xanthogranuloma (AAPOX) is a rare non-Langerhans histiocytosis characterized by periocular infiltration of foamy histiocytes, Touton giant cells, and benign hyperplasia with plasma cell infiltration in eyelids or lymph nodes. A rare variant of IgG4-RD characterized by eyelid xanthelasmas and adult-onset asthma with salivary and lacrimal glands enlargement has also been reported in the literature (88, 89).

Several observations indicate a link between IgE, IgG4 and eosinophil levels in patients with severe allergic airway disease. In a recent study of patients with difficult-to-treat asthma, elevated blood IgG4 levels were associated with higher blood IgE and eosinophil levels (90). In the same study, allergic broncho-pulmonary aspergillosis (ABPA) or eosinophilic granulomatosis with polyangiitis (EGPA) were more frequently diagnosed in patients with elevated serum IgG4 compared to the control group with normal IgG4 levels. Moreover, anti-*Aspergillus fumigatus* IgG4-antibodies were found in cystic fibrosis patients with ABPA (91). Thus, prominent IgG4 production appears to be a feature of both IgG4-RD and severe allergic airway disease.

### Association of IgG4-RD With Chronic Rhino-Sinusitis

In a recent study of IgG4-RD patients, those with chronic rhino-sinusitis (CRS) (30/46 or 65.2%) were more prone to allergic manifestations (asthma, drug allergy or allergic skin conditions) and more often had eosinophilia compared to patients without CRS (92). IgG4-related CRS is considered a specific disease entity, despite lack of consensus regarding classification/diagnostic criteria. Unlike typical CRS, IgG4-related CRS can present with bony and soft tissue invasion (93–95). Piao et al. reported that the number of IgG4-positive plasma cells/high power field (HPF) in nasal mucosa biopsies from IgG4-related CRS patients was significantly higher than in nasal biopsies from patient with granulomatosis with polyangitis (GPA) or Rosai-Dorfman disease (RDD), but not patients with fungal rhinosinusitis (FRS). In the same study, the ratio of IgG4/IgG-positive plasmacytes in nasal mucosa biopsies was >40% in 90% of IgG4-related CRS patients compared to only 10% of GPA patients, 20% of RDD patients, and 20% of FRS patients. Thus, while IgG4+ B cell infiltration is prominent in IgG4-related CRS, it is also observed in other conditions of chronic upper airway inflammation (96). There are also reported cases of isolated paranasal IgG4-RD causing destructive disease (97, 98) and periorbital IgG4-RD occurring with aspirin-exacerbated respiratory disease (AERD) (99).

### Association of IgG4-RD With Eosinophilic Granulomatosis With Polyangiitis

Eosinophilic granulomatosis with polyangiitis (EGPA) is a necrotizing vasculitis of small to medium-sized vessels associated with asthma, eosinophilia, paranasal sinusitis, pulmonary infiltrates and mononeuritis multiplex (100). Elevated serum IgG4 levels have been reported in patients with EGPA (101–103). A case of EGPA complicated by chronic symmetrical dacryoadenitis suggesting Mikulicz’s disease (a form of IgG4-RD) was also reported (104). Overall, the observations suggest shared immunopathology in EGPA and IgG4-RD.

### Association of IgG4-RD With Hypocomplementemic Urticarial Vasculitis

Mast cells are key effector cells in urticaria and many cases of angioedema (105). Histamine release from human mast cells, triggered by cross-linking of specific bound IgE to the high affinity IgE receptor, FcϵR1, is central to histamine-induced angioedema – a disorder that belongs to the spectrum of Type 2 immune diseases (106, 107) (**Table 1**). Hypocomplementemic urticarial vasculitis (HUV) is an immune-complex mediated disorder associated with urticaria and leukocytoclastic vasculitis, angioedema, arthritis, and recurrent abdominal pain (108). One proposed mechanism for urticaria and angioedema in HUV involves the formation of immune complexes, subsequent activation of the classical complement system, and generation of anaphylotoxins (e.g., C3a and C5a) that cause mast cell degranulation and histamine release (109).

High levels of serum IgG4 and an elevated IgG4/total IgG ratio were observed in one patient with typical HUV (110). Takao et al. reported a case of HUV with organ manifestations suggestive of IgG4-RD, including interstitial nephritis, and submandibular and inguinal lymphadenopathy with 40% IgG4-expressing B cells (111).

## Type 2 Immunity vs. Allergy in IgG4-RD

Even though Type 2 immune responses and allergy are observed in patients with IgG4-RD, they are not necessarily always linked mechanistically (112). Indeed, non-type 2 factors such as IgG1 immunocomplexes and complement activation system have also a role in both allergy and IgG4-RD (113–115) (**Table 1**). For example, asthma and chronic rhinitis can be caused by neutrophil- driven inflammation (116, 117), which may underlie the high prevalence of rhinosinusitis in patients with certain autoinflammatory diseases (118, 119). Another example is the association of inflammasome activation with eczema (120). These examples raise the possibility that allergy-associated phenotypes in IgG4-RD may be due to non-Type 2 immune responses but rather other types of signaling pathways.

Peripheral eosinophilia and elevated serum IgE levels have been reported along with history of allergies and atopic symptoms in 40% of patients with type I autoimmune pancreatitis, consistent with the hypothesis that allergic mechanisms drive IgG4-RD (121). However, elevated IgE levels and eosinophilia are also present in non-atopic patients with IgG4-RD kidney disease (122). Among patients with IgG4-RD in another study, the mean IgE concentrations and eosinophil blood counts were similar, regardless of history of atopy (123). Thus, IgG4-RD and allergy both appear to engage similar Type 2 pathways of immune activation, their relationships to each other in terms of pathogenesis remain uncertain.

## Therapeutic Implications and Future Directions

IgG4-RD is thought to result from abnormal activation of both innate and adaptive immunity. IgG4-RD might reflect an individual’s propensity towards pathologic Th2 immune responses, as observed in allergic conditions. Thus, like with allergy, targeting signaling pathways that are involved in Th2 immunity could be effective in IgG4-RD (**Figure 1** and **Table 2**).

**Table 2:** Select monoclonal antibodies with therapeutic potential in IgG4-related disease (IgG4-RD).

mAb	Antibody type	Target	Current Indications
Dupilumab	Fully human, IgG4	IL-4Rα; Inhibits signaling of IL-4 and IL-13	Atopic dermatitis (127, 128)
			Asthma (131)
Lebrikizumab	Humanized, IgG4	IL-13; prevents formation of IL-13Rα1/IL-4Rα heterodimer receptor signaling complex	Asthma (133)
			Atopic dermatitis (134)
Tralokinumab	Fully human, IgG4	IL-13; prevents binding of IL-13 to IL-13Rα1 and IL-13Rα2	Asthma (136)
			Atopic dermatitis (138)
Omalizumab	Humanized, IgG1	IgE Fc region	Asthma (141)
			Chronic urticaria (141)
			ABPA (142)
			CRS (142)
			Atopic dermatitis (142)
Mepolizumab	Humanized, IgG1	IL-5	Asthma (148)
			EGPA (149)
Benralizumab	Humanized, IgG1	IL-5R	Eosinophilic Asthma (146)
Reslizumab	Humanized, IgG4	IL-5	Eosinophilic Asthma (150)
Tezepelumab	Fully human	TSLP	Asthma (151)

ABPA, allergic bronchopulmonary aspergillosis; CRS, chronic rhinosinusitis, EGPA, eosinophilic granulomatosis with polyangiitis.

First-line treatment for IgG4-RD is glucocorticoids. Rituximab and other immunosuppressive agents such as azathioprine, methotrexate or mycophenolate mofetil are recommended in recurrent or refractory disease, but relapses and flares are common even with these agents (124). Multi-organ involvement, peripheral eosinophilia, and high serum levels of IgG4 and IgE at disease onset are predictors of relapse (125). After B cell depletion with rituximab, relapses may occur in more than 50% of IgG4-RD patients (126). Thus, there is a pressing need for more effective and safer therapies for patients with IgG4-RD.

Monoclonal antibodies (mAbs) targeting IL-4Ra, such as dupilumab (127), ameliorate atopic dermatitis by blocking both IL-4 and IL-13 signaling (128). In patients with IgG4-RD, this blockade could reduce inflammation and fibrosis (129). Indeed, dupilumab was able to control inflammation and fibrosis in a patient with both atopic dermatitis and IgG4-RD (130). The use of dupillumab warrants more prospective studies in IgG4-RD. Dupilumab was also recently approved for treatment of moderate to severe asthma both in adolescents and adults (131).

Kasaian et al. created a recombinant bifunctional IL-4/IL-13 antagonist that in a mouse of model of asthma reduced not only IL-4–dependent increase in serum IgE levels but also IL-13–dependent airway hyper-responsiveness and lung inflammation (132). Dual IL4/IL-13 antagonists might be particularly effective for treating patients with IgG4-RD and elevated IgE levels and/or concurrent asthma.

Two anti-IL-13 agents, lebrikizumab and tralokinumab, also have potential therapeutic activity in IgG4-RD. Lebrikizumab is a humanized IgG antibody that binds to IL-13, neutralizing its activity and improving lung function in asthma patients (133). It also appeared to be a promising therapy for moderate to severe atopic dermatitis (134). In a recent phase 2 randomized, double blind, placebo controlled trial, lebrikizumab did not show benefit in patients with idiopathic pulmonary fibrosis (IPF), either as monotherapy or in combination with pirfenidone (135). Tralokinumab prevents IL-13 binding to both IL-13Rα1 and IL-13Rα2, which are considered important mediators of Th2-related fibrosis *via* stimulation and activation of TGF-β1 (136, 137). Treatment with tralokinumab successfully improved symptoms in patients with asthma (136) and atopic dermatitis (138). Tralokinumab blockade of IL-13 attenuated lung fibrosis in a humanized mouse model of IPF (139). However, in a phase 2 randomized controlled study, tralokinumab showed no efficacy in patients with IPF (140).

Omalizumab is an anti-IgE mAb that is approved for severe allergic asthma and chronic urticaria (141). There have been many reports of off-label uses of omalizumab in diseases where IgE might have a pathogenic role, such as allergic rhinitis, ABPA, anaphylaxis, angioedema, non-atopic asthma, atopic dermatitis, EGPA, and CRS (142). By dissociated pre-bound IgE from FcϵRI, omalizumab reduces activation of mast cells and basophils (143) – a suspected mechanism of fibrosis in IgG4-RD. While omalizumab has not been evaluated for the treatment of IgG4-RD in clinical trials, it could prove to be an effective therapy, particularly in those with elevated IgE levels (**Figure 1**). However, in IgG4-associated eosinophilic esophagitis, omalizumab failed to reduce either tissue eosinophils or symptoms, despite the frequent presence of IgE bearing mast cells in esophageal tissues (144). Thus, mast cell/basophil activation may not be central to symptoms in this form of IgG4-RD.

mAbs targeting IL-5 (e.g., mepolizumab and reslizumab) or the IL-5 receptor (IL-5R) (e.g., benralizumab) reduce peripheral eosinophilia (145) and therefore might be effective therapies in IgG4-RD patients with peripheral or tissue eosinophilia. Benralizumab might be particularly effective in this regard, because it both depletes IL-5R-expressing eosinophils (through antibody-dependent cell-mediated cytotoxicity, ADCC) and limits IL-5 signaling in multiple cell types (146). Interestingly, in at least one patient with EGPA, successful treatment with benralizumab also resulted in reduction of serum IgG4 levels (147). Mepolizumab is approved for treatment of severe eosinophilic asthma and EGPA, reducing the number of eosinophils in both the blood and sputum (148, 149). Reslizumab is effective in reducing eosinophils in patients with asthma and was recently approved for the management of severe eosinophilic asthma (150).

Tezepelumab is a fully human anti-TSLP mAb that prevents interaction of TSLP with its receptor, thereby inhibiting downstream inflammatory pathways; tezepelumab has been used for the treatment of severe asthma in phase II trials (151). Combined treatment of tezepelumab and subcutaneous cat immunotherapy (SCIT) significantly decreased nasal symptoms in patients during a nasal cat allergen challenge compared to patients who received SCIT and placebo at weeks 25 and 52, but the response was not sustained after therapy was completed (152). Targeting of TSLP-mediated signaling pathway, and resultant abrogation of Th2 cascades, might be another attractive therapeutic strategy to treat IgG4-RD (**Figure 1**).

## Conclusions

IgG4-RD is a rare fibro-inflammatory disease of unknown etiology. Although the adaptive arm of the immune response is thought to play a major role in disease pathogenesis, the specific mechanisms are incompletely understood. In this review article, we discuss clinical and mechanistic overlap between IgG4-RD and allergy in order to better understand IgG4-RD and identify therapeutic targets for which approved agents already exist. A significant subset of patients with IgG4-RD have allergic disease or features of allergy, such as elevated IgE levels, tissue and blood eosinophilia, and infiltration of affected tissues by IgE-primed mast cells. More research into this subset, including genetic studies, is needed to better understand IgG-RD’s relationship to allergy.

Recent work in IgG4-RD has identified allergy-like, Th2-related pathways of aberrant immune activation that may contribute to tissue inflammation and fibrosis. These include overproduction of molecules involved in Th2 differentiation (IL-13, TSLP, IL-33), generation of IgG4- and IgE-secreting plasma cells (BAFF, IL-4, IL-13), recruitment/activation of tissue eosinophils (IL-5) and mast cells (IL-9), and tissue fibrosis (TGF-β). Thus, targeting one or more of these molecules with drugs currently available for the treatment of allergy may be beneficial in patients with IgG4-RD, for which no specific therapy has yet been proven effective.

## Author Contributions

DM, DS, TM, and GH substantially contributed to this review with regards to content and structure of the manuscript. All authors contributed to the article and approved the submitted version.

## Funding

DM is supported by Pfizer US Pharmaceuticals Group grant with sponsor award number 53857367. TM is supported by NIH grants R21AR075134, R01 AR074939 and R21 AR077266. DS is funded by NIAID intramural program. The funder bodies were not involved in the study design, collection, analysis, interpretation of data, the writing of this article or the decision to submit it for publication.

## Conflict of Interest

TM has received consulting fees from Cugene, Kiniksa, Miro Bio, and QiLu Pharmaceuticals, has an ownership share in Amdax, and has received research funding from Gilead Sciences.

The remaining authors declare that the research was conducted in the absence of any commercial or financial relationships that could be construed as a potential conflict of interest.
